# Canonical Wnt/β-Catenin Regulation of Liver Receptor Homolog-1 Mediates Pluripotency Gene Expression

**DOI:** 10.1002/stem.502

**Published:** 2010-10-23

**Authors:** Ryan T Wagner, Xueping Xu, Fei Yi, Bradley J Merrill, Austin J Cooney

**Affiliations:** a Department of Molecular and Cellular Biology, Baylor College of MedicineHouston, Texas, USA; b Department of Biochemistry and Molecular Genetics, University of Illinois at ChicagoChicago, Illinois, USA

**Keywords:** ES cells, Pluripotency, β-catenin, Lrh-1, Oct4

## Abstract

Delineating the signaling pathways that underlie ESC pluripotency is paramount for development of ESC applications in both the research and clinical settings. In culture pluripotency is maintained by leukemia inhibitory factor (LIF) stimulation of two separate signaling axes: Stat3/Klf4/Sox2 and PI3K/Tbx3/Nanog, which converge in the regulation of Oct4 expression. However, LIF signaling is not required in vivo for self-renewal, thus alternate signaling axes likely mediate these pathways. Additional factors that promote pluripotency gene expression have been identified, including the direct regulation of Oct4 by liver receptor homolog-1 (Lrh-1) and β-catenin regulation of Nanog. Here, we present genetic, molecular, and pharmacological studies identifying a signaling axis in which β-catenin promotes pluripotency gene expression in an Lrh-1-dependent manner. Furthermore, Lrh-1 was identified as a novel β-catenin target gene, and Lrh-1 regulation is required for maintaining proper levels of Oct4, Nanog, and Tbx3. Elucidation of this pathway provides an alternate mechanism by which the primary pluripotency axis may be regulated in vivo and may pave the way for small molecule applications to manipulate pluripotency or improve the efficiency of somatic cell reprogramming. Stem Cells 2010;28:1794–1804

## INTRODUCTION

The Oct4/Sox2/Nanog pluripotency axis has consistently been reiterated as the central pathway mediating ESC pluripotency and self-renewal and more recently in reprogramming somatic cells into induced pluripotent stem cells in both the mouse and human model system [1–5]. Standard methods for maintaining mouse ESC (mESC) self-renewal require supplementing media with the growth factor leukemia inhibitory factor (LIF), which acts through the receptor gp130, to stimulate two parallel pathways: Stat3/Klf4/Sox2 and PI3K/Tbx3/Nanog [6]. However, recent findings report that mESC have an innate program directing self-renewal independent of extrinsic factors, and that suppression of differentiation cues is sufficient to maintain pluripotency [7]. This finding is consistent with genetic models, which report that LIF-signaling is dispensable for self-renewal in vivo [8,9]. Similarly, LIF stimulation does not support self-renewal in human ESC culture [10]. This suggests that alternate mechanisms regulate these signaling axes in vivo. Several “secondary pluripotency factors” have been identified which are thought to be important for regulating ESC self-renewal, as well as maintaining stem cell identity during development [11,12]. Among them, the nuclear receptors Esrrβ/Nr3b2, Dax-1/Nr0b1, and liver receptor homolog-1 (Lrh-1)/Nr5a2 have been reported to comprise secondary pluripotency regulation, however, these studies are limited in that they focus on Oct4 regulation alone or do not report a clear mechanism [11–14].

Previously we reported that the orphan nuclear receptor, Lrh-1, is essential for maintaining Oct4 expression at the epiblast stage of embryonic development, and that genetic ablation causes embryonic lethality around 6.0–7.0 days postcoitum (dpc) [12]. This was supported by observations in mESC that Lrh-1 promotes Oct4 expression directly by binding evolutionary conserved elements in the proximal promoter (PP) and proximal enhancer (PE). However, Lrh-1 is not required for formation of the inner-cell mass, and Lrh-1^-/-^ ESC maintain expression of Oct4. Thus, Lrh-1 is not essential for maintaining ESC self-renewal, but rather is principal in regulating Oct4 expression during early differentiation [12].

Others have implicated Lrh-1 as a central factor governing the self-renewal of intestinal crypt cells by forming a complex with β-catenin [15]. The ubiquitous Wnt signaling pathway plays myriad roles in development and disease [16]. Canonical Wnt signaling drives self-renewal in the gut and the hematopoietic system making it an enticing mechanism to study in regard to ESC self-renewal [17–19]. Similar to the phenotype observed in the *Lrh-1* null mice, β-*catenin* null mice fail to gastrulate and die at approximately E6.0 [20]. β-catenin is not required for formation of the inner-cell mass, but is reportedly important in specifying cell fate in the pregastrulation embryo [20,21]. β-catenin is known to mediate differentiation, namely brain formation and mesoderm specification [20–24], however, several studies report a role for Wnt signaling in maintaining pluripotency [25,26]. Among them, the observation that stabilizing β-catenin through inhibition of glycogen synthase kinase-3 (GSK3) using the small molecule inhibitor 6-bromoindirubin-3′-oxime (BIO) is sufficient to maintain self-renewal in both mouse and human ESC [27]. In support of this is the finding that β-catenin promotes pluripotency by forming a complex with Oct4 that drives Nanog expression, and that stabilized β-catenin permits LIF-independent self-renewal [28].

To investigate definitively the role of canonical Wnt signaling in the regulation of pluripotency, we generated β-*catenin*^-/-^ ESC. Although exhibiting defects in differentiation, the β-*catenin*^-/-^ ESCs maintain expression of pluripotency genes, albeit at decreased levels, and serve as a stringent control for molecular studies. Among those, we observe that Lrh-1 expression is induced on stimulation of Wnt signaling using molecular and pharmacological methods, and induction occurs in a β-catenin-dependent manner. Furthermore, Wnt induction of pluripotency factors is both β-catenin and Lrh-1 dependent. We report that β-catenin regulates Lrh-1 directly via conserved lymphoid enhancer factor (LEF)/T-cell factor (TCF) sites in the full-length and embryonic stem (ES) specific promoters of *Lrh-1* [29], allowing for subsequent Lrh-1 regulation of *Tbx3*, *Nanog*, and *Oct4*. Finally, ectopic expression of Lrh-1 in β-*catenin*^-/-^ ESC is sufficient to restore Oct4 and Nanog expression to *wt* levels.

By implementing a genetic approach in the ESC model, we have revealed a secondary pluripotency axis driven by canonical Wnt regulation of Lrh-1. Elucidation of this pathway extends our bourgeoning understanding of the molecular signature of pluripotency, and may prove applicable to reprogramming and other ESC applications.

## MATERIALS AND METHODS

### Derivation of β-Catenin^-/-^ ES Cells

**Figure 1:**
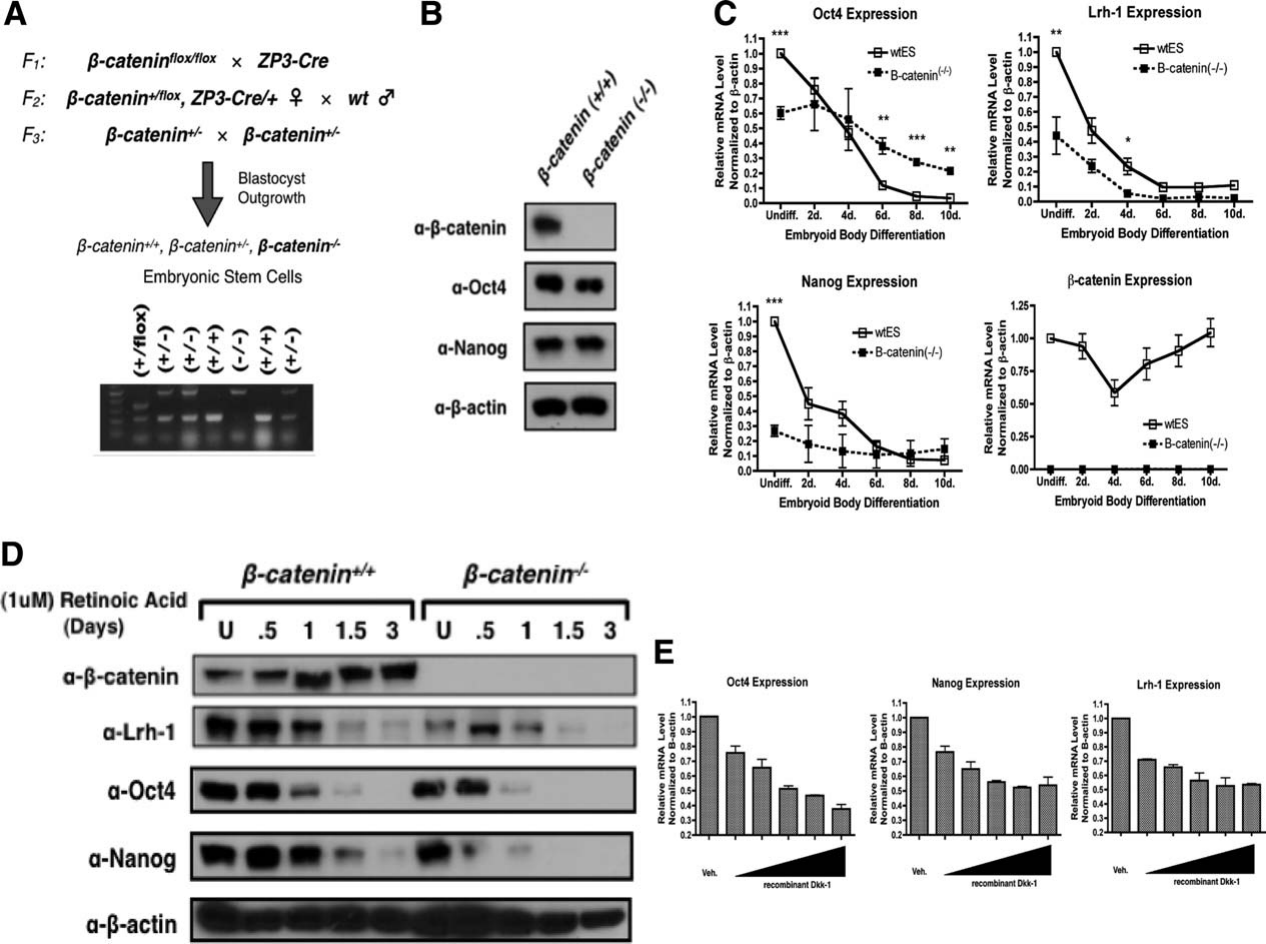
β-*catenin* promotes pluripotency gene expression mouse ESC. (**A**): Breeding schematic for the generation of β-*catenin*^±^ mice and β-*catenin*^-/-^ ES cells. Embryos were isolated at E3.5 from a heterozygote cross and individually cultured in vitro, so ES cell lines could be derived by blastocyst outgrowth. (**B**): Western blot analysis for the expression of β-catenin, Oct4, and Nanog in undifferentiated *wt* and β-*catenin*^-/-^ ESC. β-actin was probed as a loading control. (**C**): qRT-PCR analysis of Oct4, Nanog, Lrh-1, and β-catenin expression in *wt* and β-*catenin*^-/-^ ESC on embryoid body differentiation. *, *p* < .05; **, *p* < .01; ***, *p* < .001. (**D**): Western blot analysis of Oct4, Nanog, Lrh-1, and β-catenin expression in *wt* and β-*catenin*^-/-^ ESC on differentiation with retinoic acid. β-actin was probed as a loading control. (**E**): qRT-PCR analysis of Oct4, Nanog, and Lrh-1 expression in *wt* ESC on inhibition of the canonical Wnt signaling pathway with increasing concentration (1 ng–250 ng/ml) of recombinant Dkk-1 in the absence of leukemia inhibitory factor. Abbreviation: qRT-PCR, quantitative reverse transcribed-polymerase chain reaction; wt ES, wild type embryonic stem.

β-*catenin*^*flox/flox*^ male mice (Jackson Laboratories, Bar Harbor, ME, http://www.jax.org) were crossed with transgenic females expressing Cre-recombinase driven by the Zona Pelucida protein-3 (*ZP3*) promoter (Jackson Laboratories) triggering an early recombination event [22,30–32]. β-*catenin*^+/flox^, *ZP3-Cre*/^+^ females were derived and backcrossed to *wt* males to yield β-*catenin*^-/+^ offspring. Resultant β-*catenin*^-/+^ offspring were isolated and maintained in a colony (Fig. 1A). β-*catenin* heterozygote mice were bred and blastocysts isolated at 3.5 dpc. ESC lines were derived by blastocyst outgrowth as previously described [12].

### Genotyping of ES Cell Lines and Embryos

DNA was extracted from ES cell lines and mouse tails after overnight digestion in lysis buffer (0.5% sodium dodecyl sulfate, 50 mM Tris pH 7.5, 0.1 M NaCl, 5 mM EDTA, 0.2 mg/ml proteinase K), followed by phenol/chloroform/isoamyl alchohol extraction (Invitrogen, Carlsbad, CA, http://www.invitrogen.com). Genotyping was performed using the following specific primers for the β-*catenin* locus: RM41: 5′-AAGGTGGAGTGATGAAA- GTTGTT-3′, RM42: 5′-CACCATGTCCTCTGTCTATTC-3′, and RM43: 5′-TACA CTATTGAATCACAGGGACTT-3′ [22].

### Cell Culture, In Vitro Differentiation, and Small Molecule Treatments

ES cell lines were maintained on plates treated with 0.1% gelatin (Sigma, St. Louis, MO, http://www.sigmaaldrich.com), under standard conditions described previously [12]. ES cell lines were differentiated by withdrawal of LIF from ESC media and addition of 1 μM *all-trans*-retinoic acid (RA; Sigma). Embryoid body differentiation was carried out by forming the aggregates in low-attachment plates (25,000 cells/ml) or by hanging drop (500 cells/20 micro l drop) for 3 days prior to transferring the embryoid bodies to gelatinized plates. ESC lines were treated with 2 micro M BIO (Calbiochem, Darmstadt, Germany, http://www.emdchemicals.com) reconstituted in dimethyl sulfoxide (DMSO) or 100 ng/ml mWnt3a (R&D Systems, Minneapolis, MN, http://www.rndsystems.com/). Recombinant Dickkopf-1 (Dkk-1; R&D Systems) was added at increasing concentrations from 1 ng/ml to 250 ng/ml. CHIR99021 (Stemgent, San Diego, CA, http://www.stemgent.com) was added at an increasing concentration (10 nM, 100 nM, 1 micro M, 2.5 micro M, and 5 micro M). All treatments were performed in the absence of LIF.

### Transient Transfection and Plasmids

The Lrh-1 promoter regions were generously provided by You-Hua Xie [29] and were transfected alongside the TOP-FLASH reporter (Upstate, Billerica, MA, http://www.millipore.com) under the following conditions: 125,000 ESCs were transfected in suspension with 100 ng of reporter, 2 ng of *Renilla* luciferase expression vector (Promega, Madison, WI, http://www.promega.com), using 2.5 micro l Lipofectamine 2000 (Invitrogen), and plated in single well of a 12-well plate. Each transfection was assayed in triplicate. Promoter activity was measured using a Berthold Centro LB960 dual-luciferase luminometer and recorded in relative light units after normalizing to *Renilla* luciferase.

### Immunofluorescence and Western Blot

Antibody information and conditions are listed in (Supporting Information Table 1). Western Blot analysis was performed under standard denaturing conditions.

### Quantitative Reverse Transcription Polymerase Chain Reaction (qRT-PCR) Analysis

Total RNA was extracted from ES cells using Trizol reagent (Invitrogen). cDNA was generated using the Super Script III First Strand Synthesis Kit (Invitrogen) with Oligo dT primers following the manufacturer's protocol. Quantitative expression of endogenous genes was carried out using QuantiFast SYBR Green PCR (Qiagen, Valencia, CA, http://www.qiagen.com) on a Step 1 Plus Real Time PCR System (Applied Biosystems, Carlsbad, CA, https://products.appliedbiosystems.com). Target gene expression was normalized to β-actin expression in all experiments. For gene-specific primers see Supporting Information Table 2.

### Chromatin Immunoprecipitation

Chromatin immunoprecipitation (ChIP) assays were performed following a protocol modified from previous reports [12,33]. Undifferentiated *wt* and β-*catenin*^-/-^ ESCs were treated for 6 hours with 2 micro M BIO or differentiated for 2 days with 1 micro M RA prior to fixation with 1.1% formaldehyde. Cross-linked DNA underwent enzymatic shearing using the ChIP-IT Express Enzymatic kit (Active Motif, Carlsbad, CA, http://www.activemotif.com) following the manufacturers protocol. Lysate protein concentration was determined postshearing and lysates diluted to 0.25 micro g/micro l. For antibody information and immunoprecipitation conditions see (Supporting Information Table 1). Recovered DNA was quantitatively amplified with gene-specific primers spanning the putative LEF/TCF sites using QuantiFast SYBR Green PCR (Qiagen) and normalized to 10% input. For ChIP primer sequences see Supporting Information Table 3.

### Teratoma Formation

Teratomas were generated from *wt* and *Lrh-1*^-/-^ ESC after injection of approximately 10^6^ cells into the hind-quarters of nude SCID mice. Histological analysis was carried out on teratoma sections after standard H&E staining.

### Generation of Myc-Tagged Lrh-1 β-Catenin^-/-^ Stable Cell Line

The previously described embryonic isoform of Lrh-1 was cloned into a *pCMV-myc* expression vector (Clontech) using PCR primers: forward 5′-TCGAATTCTCATGCTGCCCA AAG TGGAG-3′ and reverse 5′-TAGTCGACTTAGGCTCTT TTGGCATGCAGCA-3′ [29]. Myc-tagged Lrh-1 was then subcloned into the *pEF1α-ORF-IRES-neo* vector described in with PCR primers: forward 5′-TCGCTAGCATGGCA TCAA TGCAGAAGCT-3′, and reverse 5′- TCGCGGCCGCTTAGG CTCTTTTGGCATGCA-3′ [33]. The *pEF1α-mycLrh1(ES)-IRES-neo* vector was targeted into the β-*catenin*^-/-^ ESCs via electroporation (25 micro g linearized vector/10,000,000 cells). Heterologous recombinants were screened by selection with G418 for approximately 10 days, and clonal resistant ES colonies were isolated and expanded in culture.

### Statistical Analysis

Statistical significance was determined by performing two-tailed *t*-tests where appropriate.

## RESULTS

### Generation and Characterization of *β-Catenin^-/-^* ESC

To evaluate the role of canonical Wnt signaling in ESC pluripotency, β-*catenin*^-/-^ ESC were derived by blastocyst outgrowth following a germline Cre-lox recombination event yielding heterozygous mice [22] (Fig. 1A). Western blot analysis confirms the absence of β-catenin protein in the mutant cell line, however, Oct4 and Nanog are expressed (Fig. 1B). Although β-*catenin*^-/-^ ESC express these pluripotency factors, they are not pluripotent insofar as they exhibit pronounced defects in endoderm and mesoderm induction on embryoid body differentiation (Supporting Information Fig. 1). Furthermore, two separate attempts at forming teratomas with the β-*catenin*^-/-^ ESC yielded no detectable tumors. These findings are consistent with the phenotype of the β-*catenin*^-/-^ mouse, which exhibits a gastrulation defect in vivo [20]. Nevertheless, in culture, the program for self-renewal and the expression of pluripotency factors is intact making the β-*catenin*^-/-^ ESC a useful tool for studying canonical Wnt signaling in *wt* mESC.

Proper expression of pluripotency factors is paramount for ESC self-renewal [34], so β-*catenin*^-/-^ ESC were characterized on differentiation by embryoid body formation as well as on treatment with RA. Oct4 and Nanog exhibit significant misexpression in β-*catenin*^-/-^ ESC (Fig. 1C) and are more rapidly silenced on RA-mediated differentiation, which is apparent at the protein level (Fig. 1D and Supporting Information Fig. 2). Lrh-1 is expressed at basal levels in β-*catenin*^-/-^ ESC and is nonresponsive to RA treatment (Fig. 1D and Supporting Information Fig. 2), indicating that Lrh-1 is misregulated in the β-*catenin*^-/-^ ESC. Genetically, this casts Lrh-1 as a putative β-catenin target gene.

To support this finding, we employed a nongenetic approach to inhibit canonical Wnt signaling by treating *wt* ESC with recombinant Dkk-1. Dkk-1 is a secreted factor that prevents the association of Frizzled and Lrp5-6, a necessary step in the stabilization of β-catenin [35]. Oct4, Nanog, and Lrh-1 exhibit dose-dependent repression on treatment with Dkk-1 (Fig. 1E). The observation that Dkk-1 treatment is sufficient to repress pluripotency gene expression provides independent confirmation of the defect in pluripotency gene expression observed in the β-*catenin*^-/-^ ESC.

### Lrh-1 Is Induced in a β-Catenin-Dependent Manner

**Figure 2:**
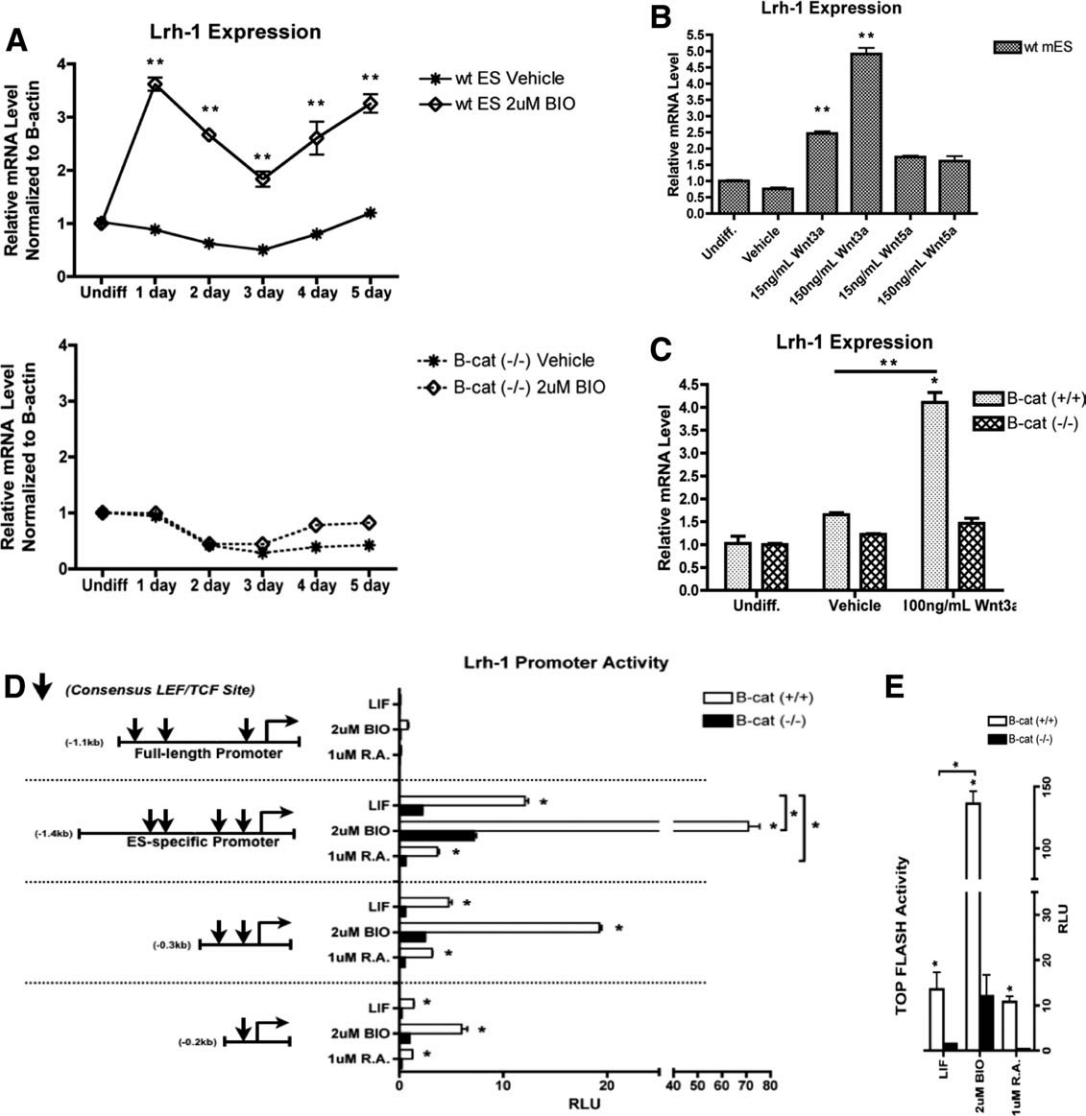
Canonical Wnt regulation of Lrh-1 expression is β-catenin dependent. (**A**): qRT-PCR analysis of Lrh-1 expression on treatment with BIO. *Wt* (upper graph) and β-*catenin*^-/-^ ESC (lower graph) treated with BIO or DMSO vehicle in the absence of LIF. **, *p* < .01. (**B**): Treatment of *wt* ESC with recombinant Wnt proteins. Lrh-1 expression was examined in undifferentiated ESC treated with increasing concentration of Wnt3a or Wnt5a in the absence of LIF. **, *p* < .01. (**C**): qRT-PCR analysis of Lrh-1 expression in *wt* and β-*catenin*^-/-^ ESC treated with recombinant Wnt3a for 24 hours in the absence of LIF. *, *p* < .05; **, *p* < .01. (**D**): Luciferase reporter assay of Lrh-1 promoter constructs. *Wt* and β-*catenin*^-/-^ ESC were transiently transfected with reporters for the *Lrh-1* full-length promoter, the *Lrh-1* ES promoter, and truncations of the ES promoter causing loss of putative LEF/TCF sites (denoted as arrows). Reporter activity was then measured in cells treated with LIF, RA, or BIO for 24 hours. *, *p* < .05. (**E**): In vitro analysis of β-catenin transcriptional activity in *wt* and β-*catenin*^-/-^ ESC using the TOP-FLASH reporter on treatment with LIF, RA, or BIO. *, *p* < .05. Abbreviations: BIO, 6-bromoindirubin-3′-oxime; DMSO, dimethyl sulfoxide; LEF, lymphoid enhancer factor; LIF, leukemia inhibitory factor; qRT-PCR, quantitative reverse transcribed-polymerase chain reaction; RA, retinoic acid; RLU, relative light unit; TCF, T-cell factor; wt ES, wild type embryonic stem; wt mES, wild type mouse embryonic stem.

In contrast to Dkk1 treatment, we sought to determine the effects of promoting canonical Wnt signaling in ESC. Promoting β-catenin stabilization with BIO, a pharmacological inhibitor of GSK3 [27], triggered a significant induction of Lrh-1 expression that was maintained for 5 days (Fig. 2A, upper graph). This effect was β-catenin-dependent, as no response was observed in the β-*catenin*^-/-^ ESC (Fig. 2A, lower graph). Additionally, β-catenin-dependent induction of Lrh-1 was recapitulated on treatment with CHIR99021, an alternate GSK3 inhibitor that is a component of the (2i/3i) GSK3/mitogen activated protein kinase Kinase (MEK) inhibitor cocktails that are reported to drive ground state pluripotency independent of LIF [7] (Supporting Information Fig. 3).

The moderate specificity that BIO has for GSK3 and the fact that GSK3 has other targets besides β-catenin led us to seek additional evidence to implicate canonical Wnt signaling in Lrh-1 induction [36,37]. Wnt stimulation was investigated as well by supplementing media with recombinant Wnt3a and Wnt5, regarded as “canonical” and “noncanonical” Wnts, respectively [38,39]. Although Wnt3a triggered a significant dose-dependent induction of Lrh-1, the noncanonical Wnt5a yielded only a modest induction that was not dose-dependent (Fig. 2B). Furthermore, Lrh-1 is induced in a β-catenin-dependent manner on stimulation with Wnt3a confirming that Lrh-1 is a novel target of the canonical Wnt signaling pathway (Fig. 2C). Wnt stimulation fully recapitulates the effects observed on GSK3 inhibition and validates BIO as a useful modulator of the pathway in our system.

### Lrh-1 Is a Direct Target of β-Catenin in mESC

LEF/TCF transcription factors mediate Wnt induced changes in gene expression through an interaction with nuclear β-catenin [16]. The murine *Lrh-1* gene contains an embryonic stem cell specific (ES) isoform driven by an alternate promoter downstream of the full-length (FL) promoter [29]. In silico analysis of each promoter region identified multiple LEF/TCF consensus sites (A/T A/T CAAAG) within each promoter [40]. Reporter constructs spanning these regions and described previously were transfected into *wt* and β-*catenin*^-/-^ ESC to compare activity [29]. Although the *Lrh-1* FL promoter exhibits basal activity in both cell lines, the 1.4 kb ES promoter revealed significantly higher activity in *wt* cells compared with the mutant cell line, which was suppressed on differentiation with RA, and induced in a β-catenin-dependent manner on BIO treatment (Fig. 2D). Transfecting serial truncations of the ES promoter yielded decreased activity, and proved less responsive to BIO implying that the putative LEF/TCF sites identified are important for activity in vitro. The decreased activity and blunted response to BIO in β-*catenin*^-/-^ ESC closely mirrored TOP-FLASH activity [41] (Fig. 2E). Interestingly, TOP-FLASH activity is not significantly repressed on RA treatment in *wt* ESC. This is in contrast to the repressed activity observed on RA treatment in cells transfected with the 1.4 kb ES-specific Lrh-1 reporter, and may be due to promoter specific effects.

**Figure 3:**
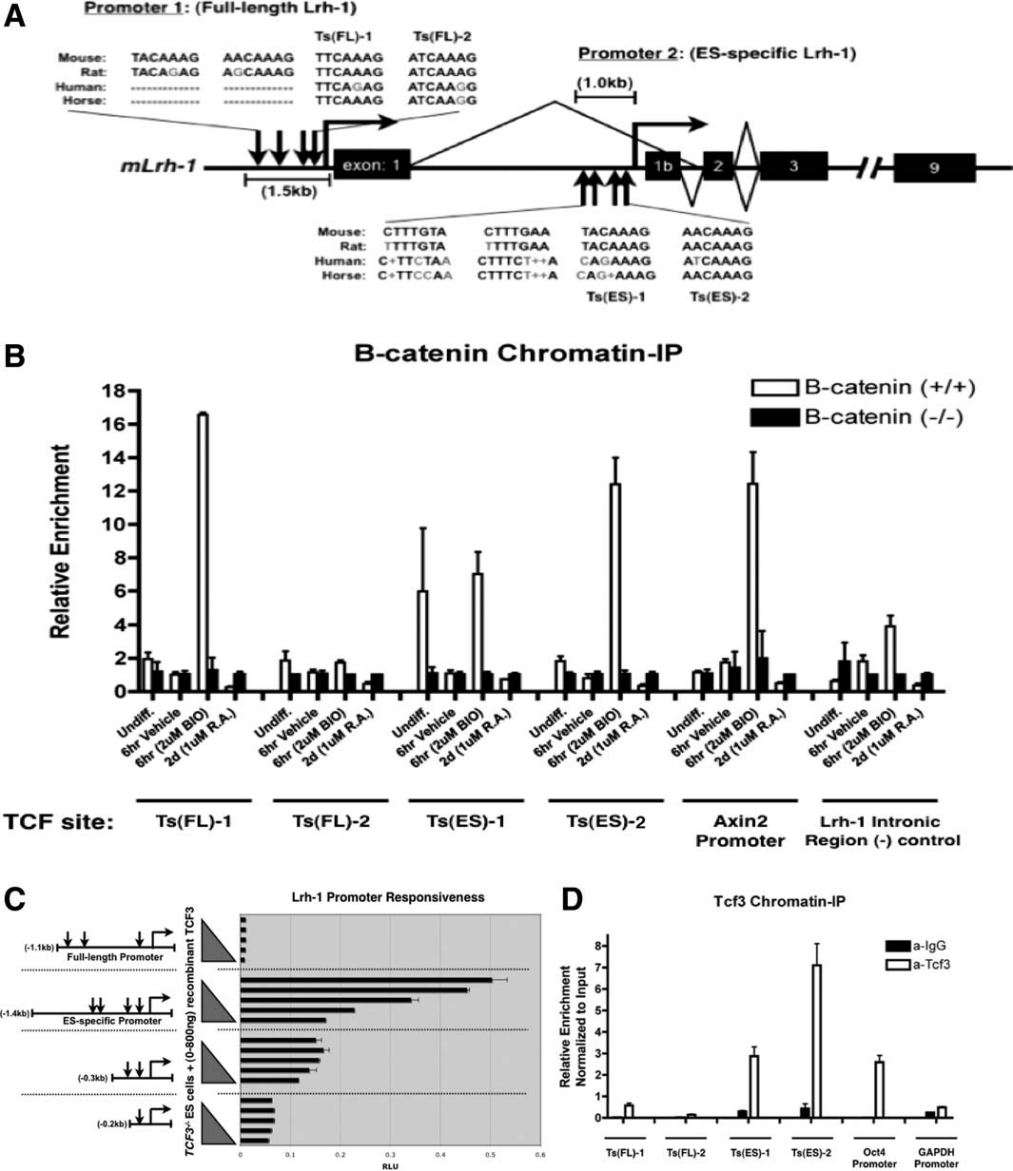
β-catenin/Tcf3 directly regulates Lrh-1 expression in mESC. (**A**): Genomic map of the *Lrh-1* regulatory regions including location and evolutionary conservation of putative LEF/TCF sites. The FL *Lrh-1* promoter contains the putative LEF/TCF sites named: Ts(FL)-1 and Ts(FL)-2, and the ES-specific promoter contains: Ts(ES)-1 and Ts(ES)-2. (**B**): Chromatin-IP (ChIP) of β-catenin. *Wt* and β-*catenin*^-/-^ ESC were treated with vehicle or BIO for 6 hours and RA for 2 days on fixation for ChIP. TCF sites: Ts(FL)-1 and Ts(FL)-2 (within the *Lrh-1* FL promoter), and Ts(ES)-1 and Ts(ES)-2 (within the *Lrh-1* ES-specific promoter) were examined for β-catenin binding. A previously reported TCF site in the *Axin2* promoter served as the positive control, and an *Lrh-1* intronic region served as the negative control. Binding in *wt* cells was normalized to the background observed in the β-*catenin*^-/-^ ESC. (**C**): Transient transfection of undifferentiated *Tcf3*^-/-^ ESC with luciferase reporters for the *Lrh-1* FL promoter, the ES promoter, and truncations of the ES promoter, in combination with increasing concentrations (0–800 ng) of *Tcf3* expression vector. (**D**): ChIP of Tcf3. Undifferentiated *wt* ESC were cultured in the presence of leukemia inhibitory factor on fixation for ChIP. Tcf3 binding was investigated at putative TCF sites: Ts(FL)-1, Ts(FL)-2, Ts(ES)-1, and Ts(ES)-2 in the *Lrh-1* promoters. The *Oct4* promoter served as positive control, and the *GAPDH* promoter served as a negative control for the assay. All treatments were normalized to input DNA and background levels for immunoprecipitation were determined by IgG precipitation. Abbreviations: BIO, 6-bromoindirubin-3′-oxime; FL, full-length promoter; GAPDH, glyceraldehyde-3-phosphate dehydrogenase; chromatin IP, chromatin immunoprecipitation; RA, retinoic acid; RLU, relative light unit; TCF, T-cell factor.

To determine if Lrh-1 induction is a primary effect of β-catenin stabilization, ChIP against β-catenin was performed. Of the eight putative LEF/TCF sites identified in the Lrh-1 promoter the highest degree of evolutionary conservation was observed at sites proximal to transcription start site in each promoter, here named: Ts(FL)-1, Ts(FL)-2, Ts(ES)-1, and Ts(ES)-2 (Fig. 3A). We therefore investigated these regions for β-catenin binding. Wild-type and β-*catenin*^-/-^ ESC were treated with BIO for 6 hours before fixation to ensure that only the immediate transcriptional effects of β-catenin would be assayed, whereas RA-treated cells were fixed after 2 days of treatment to reflect a differentiated ES cell. In the LIF-treated undifferentiated ESC, β-catenin exhibited little enrichment in both the FL and ES-specific *Lrh-1* promoter, however, LEF/TCF sites: Ts(FL)-1, Ts(ES)-1, and Ts(ES)-2 became highly enriched after treatment with BIO (Fig. 3B). β-catenin enrichment at Ts(FL-2) exhibited negligible enrichment on each treatment, implying that Ts(FL)-2 may be irrelevant in mediating β-catenin regulation of Lrh-1. Any enrichment of β-catenin is lost on RA differentiation suggesting that the direct regulation of Lrh-1 by β-catenin is not a characteristic of RA-mediated differentiation. In controls for the ChIP, enrichment of β-catenin was observed at the *Axin2* promoter after BIO treatment [42], whereas enrichment at an off-target *Lrh-1* intronic region was meager (Fig. 3B).

### β-Catenin Regulates Lrh-1 Expression Through Tcf3

β-catenin promotes target gene expression by interacting with members of the LEF/TCF family of transcription factors. Under nonstimulatory conditions, LEF/TCF factors mediate transcriptional repression of Wnt target genes through an interaction with Groucho [16]. The LEF/TCF factor, Tcf3, was previously reported to directly repress Nanog gene expression in ESC, and genetic ablation or knockdown of Tcf3 suggested that it acts as a limiter of self-renewal by counteracting effects of Nanog and Oct4 [43,44]. Others report that Tcf3 is an integral member of the Oct4/Sox2/Nanog pluripotency axis [45,46]. Considering these findings, we sought to determine if β-catenin regulation of Lrh-1 occurred through Tcf3. The *Lrh-1* reporters described in Figure 2D were transfected into undifferentiated *Tcf3*^-/-^ ESC with an increasing concentration of *Tcf3* expression vector [39]. As reported previously, the *Lrh-1* FL promoter exhibited basal activity, however, the 1.4-kb ES-specific promoter yielded high promoter activity that was repressed by Tcf3 in a concentration-dependent manner (Fig. 3C). When serial truncations of this promoter were assayed, the ability of Tcf3 to repress activity was diminished on loss of the putative LEF/TCF sites in each promoter, implying that they are necessary for mediating Tcf3 repression of Lrh-1 in vitro (Fig. 3C).

ChIP of Tcf3 exhibited enrichment at Ts(ES)-1 and Ts(ES)-2 in the *Lrh-1* ES-specific promoter, but enrichment was not observed in the FL-promoter (Fig. 3D). Tcf3 enrichment at the *Oct4* and *GAPDH* promoters was evaluated and served as positive and negative controls for the ChIP assay [45,46]. Importantly, β-catenin and Tcf3 exhibit significant enrichment at the *Lrh-1* ES-promoter, indicating a likely partnership between the two in the regulation of Lrh-1.

### Lrh-1^-/-^ ESCs Express Decreased Levels of Oct4 and Nanog

**Figure 4:**
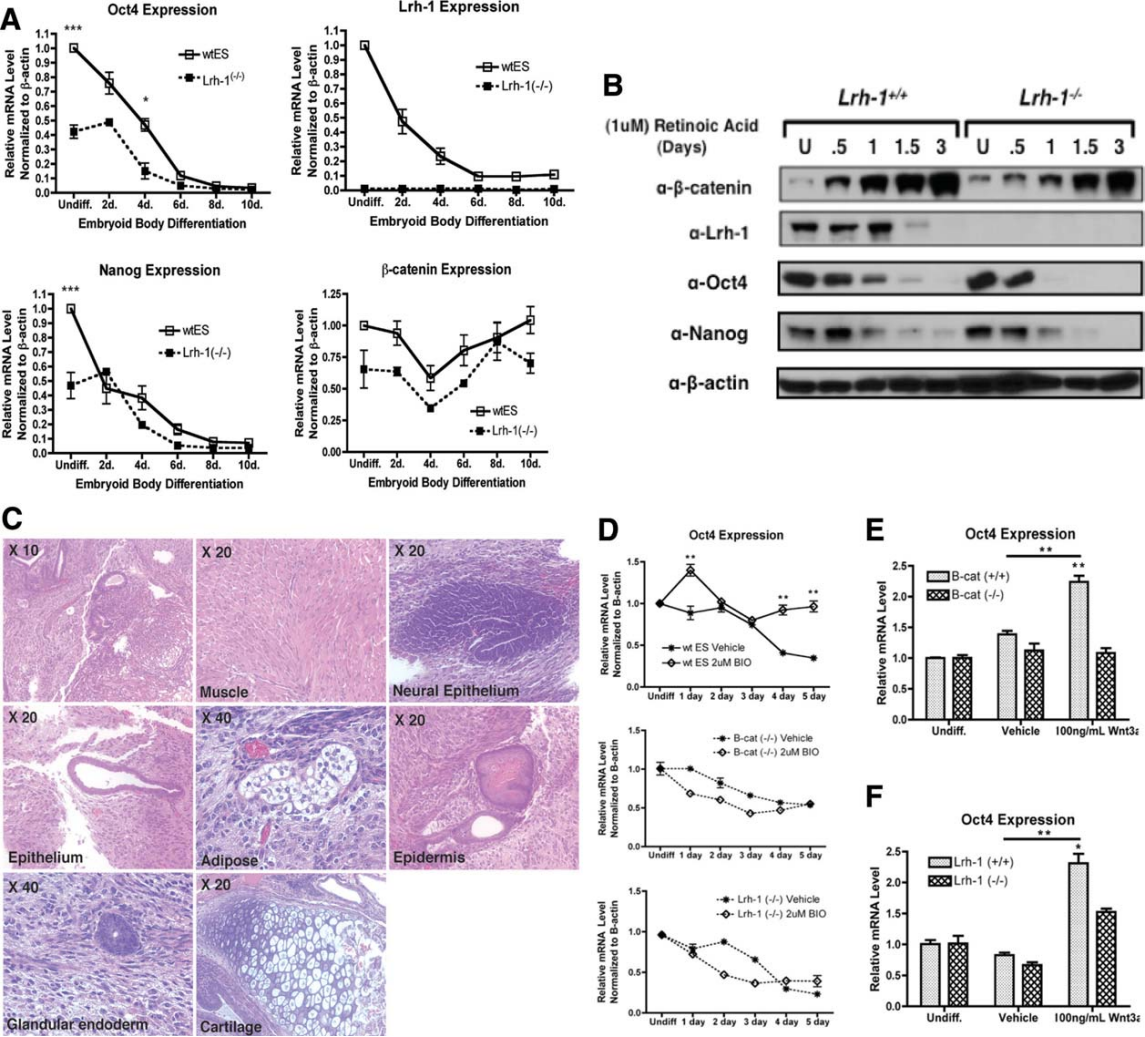
β-catenin regulation of Lrh-1 underlies the effects on Oct4 expression. (**A**): qRT-PCR analysis of Oct4, Nanog, Lrh-1, and β-catenin expression in *wt* and *Lrh-1*^-/-^ ESC on embryoid body differentiation. *, *p* < .05; ***, *p* < .001. (**B**): Western Blot analysis of Oct4, Nanog, Lrh-1, and β-catenin expression in *wt* and *Lrh-1*^-/-^ ESC on differentiation with retinoic acid. β-actin was probed as a loading control. (**C**): Histological staining of a teratoma generated by *Lrh-1*^-/-^ ESC. Endoderm tissues (epithelium, glandular endoderm), mesoderm tissues (muscle, adipose, cartilage), and ectoderm tissues (neural epithelium, epidermis) were identified. (**D**): qRT-PCR analysis of Oct4 expression on treatment with BIO. *Wt* (upper graph), β-*catenin*^-/-^ (middle graph), and *Lrh-1*^-/-^ ESC (lower graph) were treated with BIO or DMSO vehicle in the absence of leukemia inhibitory factor (LIF). **, *p* < .01. (**E**): qRT-PCR analysis of Oct4 expression on treatment of *wt* and β-*catenin*^-/-^ ESC with recombinant Wnt3a for 24 hours in the absence of LIF. **, *p* < .01. (**F**): qRT-PCR analysis of Oct4 expression on treatment of *wt* and *Lrh-1*^-/-^ ESC with recombinant Wnt3a as described in (**E**). *, *p* < .05; **, *p* < .01. Abbreviations: BIO, 6-bromoindirubin-3′-oxime; DMSO, dimethyl sulfoxide; qRT-PCR, quantitative reverse transcribed-polymerase chain reaction; wt ES, wild type embryonic stem.

If Lrh-1 is indeed a novel target gene of β-catenin in vivo, then we would expect the *Lrh-1*^-/-^ ESC to recapitulate the defect in pluripotency gene expression observed in the β-*catenin*^-/-^ ESC [12]. Wild-type and *Lrh-1*^-/-^ ESC were differentiated as embryoid bodies or by RA-mediated differentiation under the same conditions reported in Figure 1. As reported for the β-*catenin*^-/-^ ESC, the *Lrh-1*^-/-^ ESC express significantly decreased levels of Oct4 and Nanog, while expressing similar levels of β-catenin (Fig. 4A, Supporting Information Fig. 4). This defect was also apparent at the protein level in which Oct4 and Nanog underwent more rapid silencing in the *Lrh-1*^-/-^ ESC (Fig. 4B). Together, the *Lrh-1*^-/-^ ESC mirror the defect in pluripotency gene expression observed in the β-*catenin*^-/-^ ESC. In contrast to the β-*catenin*^-/-^ ESC, the *Lrh-1*^-/-^ ESC readily form teratomas composed of tissues of all three germ layers (Fig. 4C). Additionally, *Lrh-1*^-/-^ ESC can express markers of all three germ lineages on embryoid body differentiation (Supporting Information Fig. 5). In light of this finding, it seems that the gastrulation defect observed in β-*catenin*-null mice may go well beyond the misregulation of Lrh-1 alone.

### Stabilizing β-Catenin Promotes Oct4 Expression in an Lrh-1-Dependent Manner

To explore whether Lrh-1 misexpression could account for the decreased pluripotency gene expression observed in the β-*catenin*^-/-^ ESC, we assayed Oct4 expression in our cell lines after stimulating Wnt signaling with BIO or Wnt3a in the absence of LIF. BIO treatment of *wt* ESC caused a significant induction of Oct4 and was able to maintain undifferentiated levels of expression out to 5 days of treatment (Fig. 4D, upper graph). Strikingly, the BIO-mediated induction of Oct4, and subsequent maintenance of expression was observed to be both β-catenin and Lrh-1-dependent (Fig. 4D, middle and lower graphs). Despite elevated transcript levels, BIO is not sufficient to maintain Oct4 protein levels in long-term culture, which is consistent with previous reports [7] (Supporting Information Fig. 6). Treating the ES cell lines with recombinant Wnt3a yielded approximately a twofold induction in Oct4, that was entirely β-catenin-dependent (Fig. 4E). Wnt3a yielded a modest induction of Oct4 in the *Lrh-1*^-/-^ ESC, however, it was significantly less than that observed in *wt* ESC, suggesting that full induction of Oct4 requires Lrh-1 and β-catenin (Fig. 4F).

### Lrh-1 Regulates the Tbx3/Nanog Signaling Axis that Drives Self-Renewal

**Figure 5:**
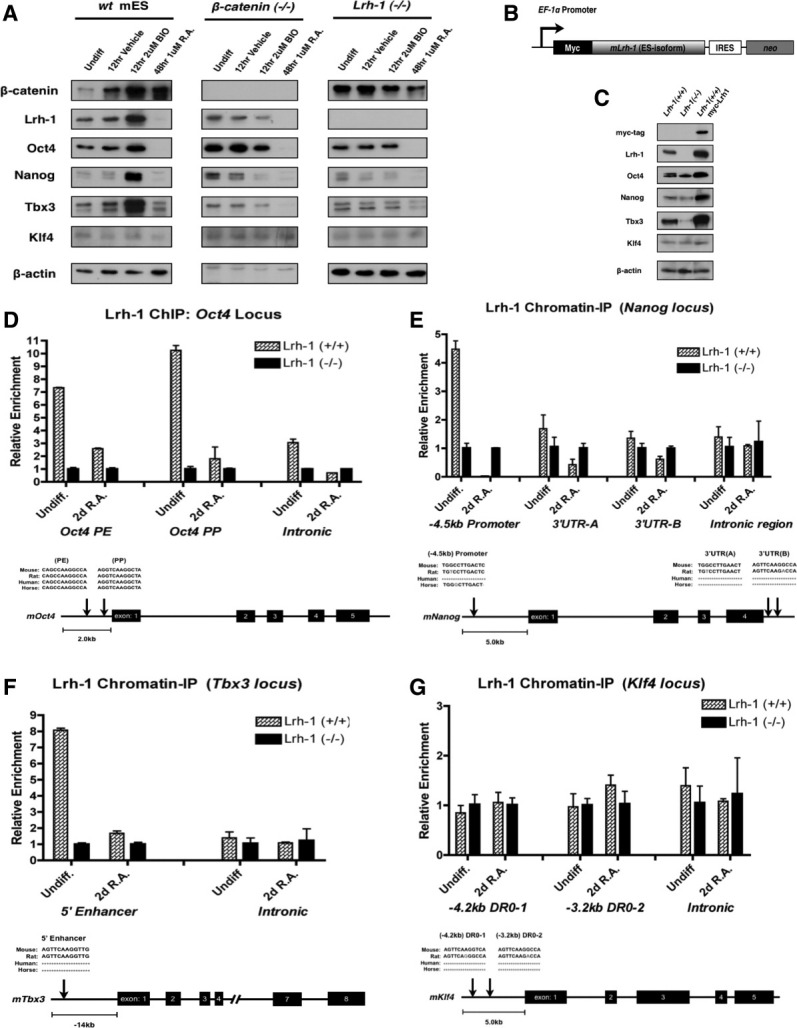
Lrh-1 directly regulates Oct4, Nanog, and Tbx3 expression in mESC. (**A**): Western blot analysis of β-catenin, Lrh-1, Oct4, Nanog, Tbx3, and Klf4 expression on inducing β-catenin stability with BIO or differentiated the ESC with RA. Wild-type, β-*catenin*^-/-^, and *Lrh-1*^-/-^ ESC were treated with DMSO vehicle or BIO for 12 hours or RA for 2 days. All treatments were performed in the absence of leukemia inhibitory factor. β-actin was probed as a loading control. (**B**): Schematic depicting the targeting vector used to generate Lrh-1 stable overexpressing ESC. Myc-tagged mLrh-1(ES)-IRES-*neo* expression is driven by the *pEF1*α promoter. (**C**): Western blot analysis of β-catenin, Lrh-1, Oct4, Nanog, Tbx3, and Klf4 expression in undifferentiated *wt*, *Lrh-1*^-/-^, and *Lrh-1^+/+^(myc-Lrh-1*) stably expressing ESC. β-actin was probed as a loading control. (**D**): Direct regulation of Oct4 by Lrh-1. ChIP experiment exhibiting direct binding of Lrh-1 to the PP and PE of the *Oct4* promoter. Binding in *wt* cells was normalized to the background observed in the *Lrh-1*^-/-^ ES cells. An intronic region was examined as a negative control for binding. (**E**): Direct regulation of Nanog by Lrh-1. Experiment and analysis was performed as in (**D**). Enrichment of Lrh-1 binding is observed in the 5 kb promoter, however, enrichment was not observed at consensus sites in the 3′UTR. (**F**): Direct regulation of Tbx3 by Lrh-1. Experiment and analysis was performed as in (**D**). Enrichment of Lrh-1 binding is observed within the enhancer region of *Tbx3*. (**G**): Lrh-1 ChIP targeting the *Klf4* promoter. Experiment and analysis was performed as in (**D**). Enrichment of Lrh-1 was not observed within the *Klf4* promoter despite the presence of two consensus sites in the Klf4 promoter. Abbreviations: BIO, 6-bromoindirubin-3′-oxime; ChIP, chromatin Immunoprecipitation; DMSO, dimethyl sulfoxide; EF-1α, elongation factor -1α; IRES, internal ribosome entry site; PE, proximal enhancer; PP, proximal promoter; RA, retinoic acid; wt mES, wild type mouse embryonic stem.

To investigate the significance of β-catenin regulation of Lrh-1 in ESC, we wanted to determine if Lrh-1 affects the Stat3/Klf4/Sox2 or Tbx3/Nanog signaling axes. In culture, LIF supports mESC self-renewal by promoting two parallel signaling axes [6]. One axis includes activation of Stat3, causing induction of Klf4, which in turn regulates expression of Sox2, whereas the parallel axis comprises activation of PI3K, causing induction of Tbx3, and subsequent regulation of Nanog expression. Intriguingly, LIF is not required in vivo to maintain pluripotency suggesting alternate mechanisms may promote these signaling axes [9]. To investigate if β-catenin regulation of Lrh-1 has a role in regulating these signaling axes, *wt* ESCs were treated with BIO to determine if stimulating Wnt signaling is sufficient to induce expression of these factors. Lrh-1, Oct4, Nanog, and Tbx3, all exhibit significant induction on BIO treatment in *wt* ESC, and are repressed on differentiation with RA (Fig. 5A). Importantly, the induction of these factors is both β-catenin-dependent and Lrh-1-dependent because neither of the mutant cell line exhibited a response to BIO. In contrast, BIO was ineffective at inducing expression of Klf4 in *wt* and mutant ESC (Fig. 5A).

To support the pharmacological data, we incorporated a genetic approach by stably overexpressing a myc-tagged ES isoform of *Lrh-1* in a *wt* mESC [33] (Fig. 5B). Overexpression of Lrh-1 results in a considerable induction of Oct4, Nanog, and Tbx3 expression above the levels observed in *wt* ESC (Fig. 5C). Additionally, these factors all exhibit decreased expression in *Lrh-1*^-/-^ ESC suggesting that Lrh-1 is required to maintain *wt* levels of expression. Consistent with BIO treatment, Klf4 expression is nonresponsive to either *Lrh-1* ablation or overexpression. ChIP of Lrh-1 was performed to determine whether Lrh-1 regulated *Tbx3* and *Nanog* expression directly. Consistent with our previous findings, Lrh-1 enrichment was observed at the *Oct4* PP and PE in ESC [12] (Fig. 5D). Lrh-1 was also enriched at a response element in the *Nanog* promoter (Fig. 5E) and in the *Tbx3* enhancer region (Fig. 5F). Consistent with the previous data, Lrh-1 was not enriched at the *Klf4* locus despite two nearly perfect consensus sites within the promoter (Fig. 5G). These data suggest that Lrh-1 directly regulates *Tbx3*, *Nanog*, and *Oct4* expression in undifferentiated ESCs, and thereby selectively promotes the Tbx3/Nanog pluripotency axis.

### Ectopic Expression of Lrh-1 Restores Oct4/Nanog Gene Expression in β-Catenin^-/-^ ESC

**Figure 6:**
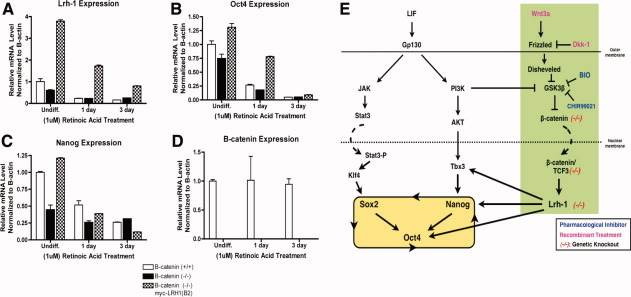
Ectopic expression of Lrh-1 restores pluripotency gene expression in β-*catenin*^-/-^ ESC. (**A**): qRT-PCR analysis of Lrh-1 expression in undifferentiated *wt*, β-*catenin*^-/-^, and β-*catenin*^-/-^(myc-Lrh-1) ESC on RA-mediated differentiation. (**B**): qRT-PCR analysis of Oct4 expression. Treatments were as described in (**A**). (**C**): qRT-PCR analysis of Nanog expression. Treatments were as described in (**A**). (**D**): qRT-PCR analysis of β-catenin expression. Treatments were as described in (**A**). (**E**): Using genetic knockout ESC models, we have shown β-catenin-dependent regulation of Lrh-1 mediates expression of pluripotency genes. This finding was observed by employing both molecular and pharmacological tools to stimulate or inhibit canonical Wnt signaling in our ESC lines. β-catenin and Tcf3 exhibit significant enrichment at the *Lrh-1* ES-specific promoter suggesting they likely partner in the direct regulation of Lrh-1 expression in the ESC. Furthermore, Lrh-1 was observed to directly regulate Oct4, Nanog, and Tbx3 expression, but not that of Klf4. Our data suggests that canonical Wnt regulation of Lrh-1 can significantly influence the PI3K/Tbx3/Nanog/Oct4 signaling axis while having little effect on the parallel Stat3/Klf4/Sox2/Oct4 signaling axis that has previously been modeled by Niwa et al. [6]. β-catenin regulation of Lrh-1 may be central to regulating the former signaling axis in vivo. Abbreviations: BIO, 6-bromoindirubin-3′-oxime; LIF, leukemia inhibitory factor; qRT-PCR, quantitative reverse transcribed-polymerase chain reaction.

To further substantiate our hypothesis that misregulation of Lrh-1 in β-*catenin*^-/-^ ESC is in part causing the defect in pluripotency gene expression, Lrh-1 was stably expressed in a β-*catenin*^-/-^ ESC as described in (Fig. 5B), in an attempt to restore *wt* levels of pluripotency gene expression (Supporting Information Fig. 7). The stable clone, β-*catenin^-/-^ myc-Lrh-1(B2)*, expressed Lrh-1 at levels fourfold higher than that observed in undifferentiated *wt* cells (Fig. 6A). Importantly, overexpression was able to restore Oct4 expression, as well as prolong expression during RA differentiation (Fig. 6B). Similarly, Nanog expression was restored to *wt* levels in undifferentiated cells (Fig. 6C). Canonical Wnt signaling was not restored in the stable cell line, which is apparent by the absence of β-catenin expression, and confirms that the observed effects on pluripotency gene expression are specific to ectopic Lrh-1 expression (Fig. 6D).

## DISCUSSION

Self-renewal of mESC in culture is directed by LIF stimulation of parallel signaling pathways (Stat3/Klf4/Sox2 and PI3K/Tbx3/Nanog) that converge in the regulation of Oct4, thereby establishing the Oct4/Sox2/Nanog autoregulatory loop [1,6]. Intriguingly, ablation of LIF signaling in the embryo at either the ligand or receptor level does not yield an early embryonic phenotype, thus LIF is not required to maintain pluripotency in vivo [8,9]. This suggests alternate mechanisms exist to promote these two signaling axes during development.

Here, we propose a secondary pluripotency axis, namely, canonical Wnt regulation of Lrh-1 resulting in direct regulation of Tbx3, Nanog, and Oct4. We believe this mechanism selectively promotes the PI3K/Tbx3/Nanog signaling axis so as to counteract germ cell nuclear factor (GCNF)-mediated repression of Oct4 [47,48]. This pathway was genetically elucidated by profiling pluripotency gene expression in β-*catenin*^-/-^ and *Lrh-1*^-/-^ ESC and determining the requirement of these factors in promoting pluripotency after stimulating Wnt signaling using various molecular and pharmacological methods (Fig. 6E). We have identified Lrh-1 as a novel direct target gene of β-catenin and Tcf3, and we identified Tbx3 and Nanog as direct targets of Lrh-1. We believe the linear nature of the results, and rescue of the β-*catenin*^-/-^ ESC by ectopic Lrh-1 expression is sufficient evidence to surmise that the decrease in pluripotency gene expression in our mutant ESC relative to *wt* ESC is not due to cell line to cell line heterogeneity alone. We present a LIF-independent mechanism in which canonical Wnt regulation of Lrh-1 selectively promotes the PI3K/Tbx3/Nanog signaling axis over the Stat3/Klf4/Sox2 axis, as Klf4 was not responsive to Lrh-1 induction or overexpression. We believe this mechanism is likely a physiologically relevant alternative to LIF-mediated pluripotency, which is known to be nonessential during early development, but rather is an evolutionary adaptation for diapause; a physiological recess in the development of the embryo induced by a lack of resources [9].

Previous reports have identified PI3K as a component of the LIF signaling cascade that functions independent of Stat3 [49]. Its role in regulating ESC pluripotency was reported to occur chiefly through GSK3 inhibition and induction of Nanog [50]. Though PI3K is known to promote β-catenin stability through GSK3 inhibition, the authors questioned the importance of β-catenin as little nuclear translocation was observed. In light of the β-catenin-dependent roles reported here, it is of future interest to determine the role of PI3K using our genetic models.

Tcf3 is reported to play an unconventional role in mediating the effects of the primary pluripotency axis by forming a complex with Oct4, Sox2, and Nanog [45,46]. Surprisingly, a genome-wide binding assay performed for Tcf3 did not identify Lrh-1 as a target gene [45]. One possibility is that the antibody used, or conditions established, may favor immunoprecipitation of a Tcf3/Oct4/Sox2/Nanog complex over a Tcf3/β-catenin complex. *Tcf3*^-/-^ ESC exhibit increased and prolonged expression of Nanog and Lrh-1 during ESC differentiation [43]. This phenotype is due to loss of Tcf3-mediated repression, and implies that Lrh-1 is a target of Tcf3 in ESC.

Recent studies have described a “ground state” to ESC pluripotency, concluding that ESC can self-renew independent of growth factors and cytokines when differentiation cues are absent, thus, ESC have an innate program directing self-renewal [7]. Whether canonical Wnt regulation of Lrh-1 plays a role in promoting ground state pluripotency is unknown, however, GSK3 inhibition is essential in driving ground state pluripotency both in ESC culture and in reprogramming [7,51]. Alternatively, canonical Wnt regulation of Lrh-1 may be a signature of a different pluripotent cell fate such as the epi-stem cell. This is supported by the fact that mutant ESC lines for β-catenin, Lrh-1, and Nanog have each been derived and can self-renew indefinitely in culture, however, in the case of Nanog, its expression is required to establish the pluripotent epiblast [51,52]. It is of future interest to investigate this signaling axis in pluripotent cell types derived at developmentally later stages. A Wnt driven mechanism promoting pluripotency gene expression, could allow for a more acute spatial and temporal response to ensure the proper balance in expression.

It is also of interest to see to what degree the β-catenin/Lrh-1 pathway can influence the emerging field of somatic cell reprogramming. iPSCs hold a great deal of promise for future therapeutic applications, however, caveats exist, such as the efficiency of reprogramming and their safety in application. Recently, another group identified Lrh-1 as being sufficient to replace Oct4 during viral-mediated reprogramming [53]. Others report that stimulation of the canonical Wnt signaling pathway with recombinant Wnt3a promotes the reprogramming of somatic cells in the absence of c-Myc, thereby circumventing a major criticism of iPS generation [54]. The discovery of nongenetic approaches to improve reprogramming, such as through the use of small molecules and signaling peptides that can be added exogenously is paramount for the field.

The intricate nature of the Wnt signaling pathway makes it amenable to small molecule manipulation. Many known modulators of Wnt signaling have been characterized, and may hold potential not just in promoting somatic cell reprogramming, but perhaps in directing differentiation into specific lineages as well [55]. Additionally, research is currently underway to identify a physiological ligand for Lrh-1, as well as develop synthetic molecules that may modulate Lrh-1 transcriptional activity. The development and characterization of such modulators may prove beneficial in a host of ESC applications and in cancer models as well.

## CONCLUSION

Here, we present evidence that canonical Wnt/β-catenin pathway regulates Lrh-1 during ESC self-renewal thereby comprising a secondary pluripotency axis in the promotion of Oct4, Nanog, and Tbx3 gene expression. We base this finding on the incorporation of genetic, pharmacological, and molecular studies that are described herein. Furthermore, Lrh-1 was identified as novel target gene of both β-catenin and Tcf3, indicating they likely partner in regulating Lrh-1 expression in the ESC. We believe this pathway to be significant as it provides an alternate mechanism to LIF-mediated pluripotency, which is known to be dispensable in vivo [8,9], and may prove amenable to small molecule applications to direct reprogramming or differentiation.

## DISCLOSURE OF POTENTIAL CONFLICTS OF INTEREST

The authors indicate no potential conflicts of interest.
